# Intra-articular injection of ascorbic acid enhances microfracture-mediated cartilage repair

**DOI:** 10.1038/s41598-024-54514-x

**Published:** 2024-02-15

**Authors:** Zhian Chen, Sihe Zhang, Peiya Duan, Zhengbo Yin, Shuangbin Dong, Rongqing Pang, Hongbo Tan

**Affiliations:** 1 Graduate School, Kunming Medical University, Kunming City, Yunnan Province China; 2 Department of Cell Biology, School of Medicine, Nankai University, Tianjin, China; 3 Neurology Department, Longling County People’s Hospital, Baoshan City, Yunnan Province China; 4 Basic Medical Laboratory, People’s Liberation Army Joint Logistic Support Force 920th Hospital, Kunming City, Yunnan Province China; 5 Department of Orthopaedics, People’s Liberation Army Joint Logistic Support Force 920th Hospital, Kunming City, Yunnan Province China

**Keywords:** Intra-articular injection, Ascorbic acid, Microfractures, Cartilage repair, Diseases, Molecular medicine, Nephrology

## Abstract

Previous studies have confirmed that ascorbic acid (AA) can promote cartilage repair and improve cartilage differentiation in bone marrow mesenchymal stem cells. However, the use of microfracture (MFX) combined with AA to repair cartilage damage has not been studied. This study established a rabbit animal model and treated cartilage injury with different concentrations of AA combined with MFX. Macroscopic observations, histological analysis, immunohistochemical analysis and reverse transcription quantitative polymerase chain reaction analysis of TGF-β, AKT/Nrf2, and VEGF mRNA expression were performed. The results showed that intra-articular injection of AA had a positive effect on cartilage repair mediated by microfractures. Moreover, 10 mg/ml AA was the most effective at promoting cartilage repair mediated by microfractures. Intra-articular injection of AA promoted the synthesis of type II collagen and the formation of glycosaminoglycans by downregulating the mRNA expression of TGF-β and VEGF. In summary, this study confirmed that AA could promote cartilage repair after MFX surgery.

## Introduction

Focal cartilage defects of the knee joint are common in orthopaedic diseases. Approximately 10% of adolescent patients have sports injuries of the knee joint accompanied by cartilage injury. The incidence rate of cartilage injuries diagnosed by arthroscopy is 57.3%^1,2^. Local focal cartilage defects exceeding 4 cm^2^ can cause serious complications and often require surgical repair^3,4^. At present, surgical treatment strategies include autologous chondrocyte implantation, autologous bone cartilage transplantation^5–7^, allogeneic transplantation^8^, cartilage tissue engineering^9–11^, and other treatments. Although these strategies are commonly used on animal models, they are not widely accepted due to the complexity of the surgical procedures and poor long-term efficacy^7^. Microfracture (MFX) stimulation of bone marrow mesenchymal stem cells (BMSCs) is still the most widely used clinical treatment^12^, but many studies have shown that the long-term efficacy of MFX is poor, mainly due to the formation of fibrocartilage rather than normal hyaline cartilage^13^. Therefore, the regeneration and clinical transformation of hyaline cartilage after cartilage injury is a major bottleneck.

In recent years, many researchers have studied alternatives to second-generation microfractures for enhancing cartilage repair, which is known as "MFX-enhanced" technology^14^. MFX technology combines cytokines, biomaterials, and chemical therapy to improve the repair of cartilage damage by stimulating the differentiation of BMSCs into chondrocytes^15–20^. Many drugs, including hormones^21,22^, monoclonal antibodies^20^, statins^23^, analgesics^24^, and nutritional supplements (glucosamine, chondroitin sulfate, high-molecular-weight hyaluronic acid, ascorbic acid (AA), vitamin D, calcium gluconate), have been shown to have beneficial effects on cartilage repair in vivo and in vitro^25–28^, and nutritional supplements are currently the most commonly used agents in clinical practice. However, the effect of the combination of AA and MFX technology on cartilage repair has not been determined.

There are three main reasons why AA (vitamin C) promotes the transformation of mesenchymal stem cells (MSCs) into transparent chondrocytes. First, AA is a natural and effective antioxidant. It can effectively eliminate reactive oxygen species (ROS) in MSCs and promote Nrf2 activity, thereby inhibiting MSC ageing and promoting the ability of these cells to proliferate and differentiate^29,30^. Second, AA is a cofactor in various enzymatic reactions and promotes MSC differentiation into chondrocytes by increasing lysine hydroxylase activity and reducing proline hydroxylase activity^31^ while stimulating collagen gene transcription and glycosaminoglycan synthesis and reducing chondrocyte apoptosis^32,33^. Third, 0.2 mM AA can effectively promote the formation of transparent cartilage^27^, and studies have shown that AA can inhibit the expression of TGF-β1 and VEGF and inhibit tissue fibrosis^15,34,35^.

To our knowledge, there have been no studies on the effect of AA combined with MFX on cartilage repair through the stimulation of bone marrow mesenchymal stem cells. Therefore, the main purpose of this study was to investigate the in vivo effects of AA and microfracture stimulation of the bone marrow mesenchymal joint on cartilage repair.

## Materials and methods

### Experimental animals

The animal experiments were approved by the Committee for Animal Use of the 920th Hospital of the PLA Joint Logistics Support Force. The methods were carried out in accordance with the relevant guidelines and regulations. All studies were performed in accordance with the ARRIVE guidelines. This study used 60 healthy New Zealand white rabbits (Kunming Kejing Co., Ltd., weight: 3 ± 0.2 kg, aged 4 months, no sex restrictions) without knee joint injury. The animals were randomly divided into 5 groups (each = 12 knees): (1) microfracture group (MFX group); (2) 1 mg/mL group (MFX + 1 mg/mL AA injection); (3) 3 mg/mL group (MFX + 3 mg/mL injection AA); (4) 10 mg/mL group (MFX + 10 mg/mL injection of AA); and (5) 30 mg/mL group (MFX + 30 mg/mL injection of AA). The rabbits were placed in a stainless steel cage with a grid at the bottom and allowed to move freely. The animals were fed pellet feed and continuously received tap water. During the experiment, strict adherence to humanitarian norms and requirements was maintained, and animal handling met humanitarian requirements. This study was approved by the Ethics Committee of the 920th Hospital of the Joint Logistics Support Force (Ethics 2022-072 (Department)-01).

### Microfracture surgery and AA injection

The left/right knees of sixty rabbits were randomly selected using a computer, and each rabbit was anaesthetized with pentobarbital sodium (3%, 1 mL/kg; Shandong Huamu Pharmaceutical Co., Ltd.). After successful anaesthesia, the hair in the surgical area of the unilateral knee joint was shaved off, the area was disinfected with 75% iodophor, and surgical dressing was placed on a single surgical area. A longitudinal incision 4–5 cm in length was made along the medial side of the patella to fully expose the femoral trochlear surface. A trephine was used to form a bone cartilage defect (diameter 5 mm, depth 2 mm) in the trochlear groove^18^. Then, immediately after the osteochondral defect was created, a drill bit with a diameter of 0.7 mm was used to drill 5 holes with a distance of 0.9 mm and a depth of 2 mm for microfractures^20^. Then, 3–0 Vicryl ® (Aixikang Co., Ltd.) sutures were used to suture the capsule and skin, After the wound was sutured, professional veterinarians used the transpatellar tendon approach to inject 1 ml AA into the joint cavity with 1 ml syringe with a 26G removable needle; the injection concentrations were 1 mg/ml, 3 mg/ml, 10 mg/ml, and 30 mg/ml^36^, and the injection concentrations were labelled on each cage.

The indicated concentration was injected into the knee joint again 2 and 4 weeks after surgery. Veterinary staff from the Animal Research Laboratory of a tertiary hospital provided standard postoperative care for the rabbits, including infection prevention, pain reduction, wound observation, and disinfection.

### General observations

During the 6th and 12th weeks after surgery, 6 rabbits (6 knees each) were randomly selected from each group and euthanized by intravenous injections of pentobarbital (3%, 100–150 mg/kg; Shandong Huamu Pharmaceutical Co., Ltd.). The distal femur was immediately dissected after euthanasia. Three observers performed gross observations in a blinded manner, and the results were evaluated using the International Society for Cartilage Repair (ICRS) scoring system^18^. The scoring system is composed of three categories (degree of defect repair, integration to the border zone, macroscopic appearance) and assigns a score ranging from 0 to 12 points; the higher the score, the better the repair.

### Imaging evaluation

After the knee joint was fixed with neutral buffered formalin for 1 week, the distal femur was wrapped in paraffin to prevent dryness during scanning. A Skyscan 1276 Micro-CT instrument (Bruker microCT, Kontech, Belgium) was used to scan the femur with the following parameters: voltage, 100 kV; current, 200 μA; AI + Cu filter; voxel size, 20 µm; and rotation step, 0.4°. Then, the image was reconstructed using NRecon software (Bruker microCT, Kontech, Belgium), and the following settings were used: the annular artefact was corrected to 5; and smooth to 3. In addition, 200 slices (4 mm) in the middle of the defect area were analysed, and the same threshold (cross-sectional view) was used to evaluate subchondral bone healing.

### Histological assessment

Knee joint tissue was decalcified with 10% EDTA and 1% sodium hydroxide for 4 weeks and was decalcified with 5% formic acid for 2 weeks. After the decalcification was complete, the sample was dehydrated through a graded alcohol series, cleared with xylene, soaked in wax, and then embedded. The paraffin was fixed on the slicing machine, the thickness of the slice was adjusted to 4 µm, paraffin slices were prepared, and the slices were dewaxed in water. A haematoxylin and eosin (HE) staining kit (G1120, Solarbio, Shanghai, China) and a safranin O staining kit (G1371, Solarbio, Shanghai, China) were used. A fluorescence microscope (Nikon Eclipse E100, Nikon, Japan) was used for microscopic examinations, and panoramic scanning was performed using an imaging system (Nikon DS-U3, Nikon, Japan) for image acquisition and analysis.

### Immunohistochemistry

Paraffin sections were dewaxed and rehydrated to water, antigenic repair was performed using repair solution, and serum was then used for closure. Next, the sections were incubated with primary antibodies against Collagen I (1:500, 14695-1-AP; San Ying Biological, Wuhan, China), Collagen II (1:500, 28459-1-AP; San Ying Biological, Wuhan, China), and Collagen III (1:400, 22734-1-AP; San Ying Biological, Wuhan, China) overnight and PBS wash away residue antibody,after that incubated with goat anti-rabbit secondary antibody conjugated with –HRP for 50 min. Then, diaminobenzidine (DAB) was used to develop the colour, and nuclear counterstaining was performed with haematoxylin. After being dehydrated, the slides were sealed with neutral gum. Finally, a fluorescence microscope (Nikon Eclipse E100, Nikon, Japan) was used to observe the sections, and an imaging system (Nikon DS-U3, Nikon, Japan) was used to perform panoramic scanning and collect the images.

### Gene expression analysis

The rabbits were euthanized 6 weeks after surgery, and patellar fat pad tissue was collected from both knee joints and stored at − 80 °C. Total RNA was extracted according to the instructions of a TRIzol reagent kit (Invitrogen, USA), and a SureScript First-strand cDNA synthesis kit (Servicebio, Guangzhou, China) was used to reverse transcribe total RNA into cDNA. The cDNA was subjected to qPCR using a 2 × Universal Blue SYBR Green qPCR Master Mix reagent kit (Servicebio, Guangzhou, China). The primer sequences are shown in Table 1. The melting curve was smooth and had only one large peak, which indicated good primer specificity and data availability. Relative gene expression was calculated with the ΔCt-Ct gene-Ct reference method, and the fold change in gene expression was calculated by the 2^−ΔΔCt^ method.

**Table 1:** Primer sequences.

Gene ID	Primer sequences
VEGF F	CCATGGCAGAAGAAGGAGA
VEGF R	ACGCAGGAAGGCTTGAATA
TGF-β1 F	TCGGTTCCTTTGATGGAC
TGF-β1 R	GTTAGAAGAGATGCGGGG
AKT F	TCCCGTCTTTCACCCACT
AKT R	GGTTCCGCCTCCACTTTG
Nrf2 F	TTTTGAGGATTCTTTCGGC
Nrf2 R	TCTGTGCTTTCAGGGTGGT
GAPDH F	GGGCGGAGCCAAAAGG
GAPDH R	GGGTGGGCACACGGAA

### Statistical analysis

The results are expressed as the mean ± standard deviation (IBM SPSS Statistics 23.0 statistical software). The positive area ratios of type I collagen, type II collagen and type III collagen were calculated using ImageJ (version 1.8.0.345). ANOVA was used for statistical analysis, followed by Tukey’s post hoc test for two-group comparisons. The data were analysed using GraphPad Prism (version 9.0). A value of *P* < 0.05 was considered to indicate statistical significance.

### Ethics statement and animal studies

The Institutional Review Board approved the research protocol. The use of all animals followed the guidelines of the Ethics Review Committee of the 920th Hospital of the PLA Joint Logistics Support Force and was approved by the Ethics Committee (2022-072 (Department) -01). All methods were carried out in accordance with relevant guidelines and regulations. All studies were performed in accordance with the ARRIVE guidelines.

## Results

### Gross observations, ICRS scores and micro-CT evaluations of subchondral bone healing 6 weeks after surgery

**Figure 1:**
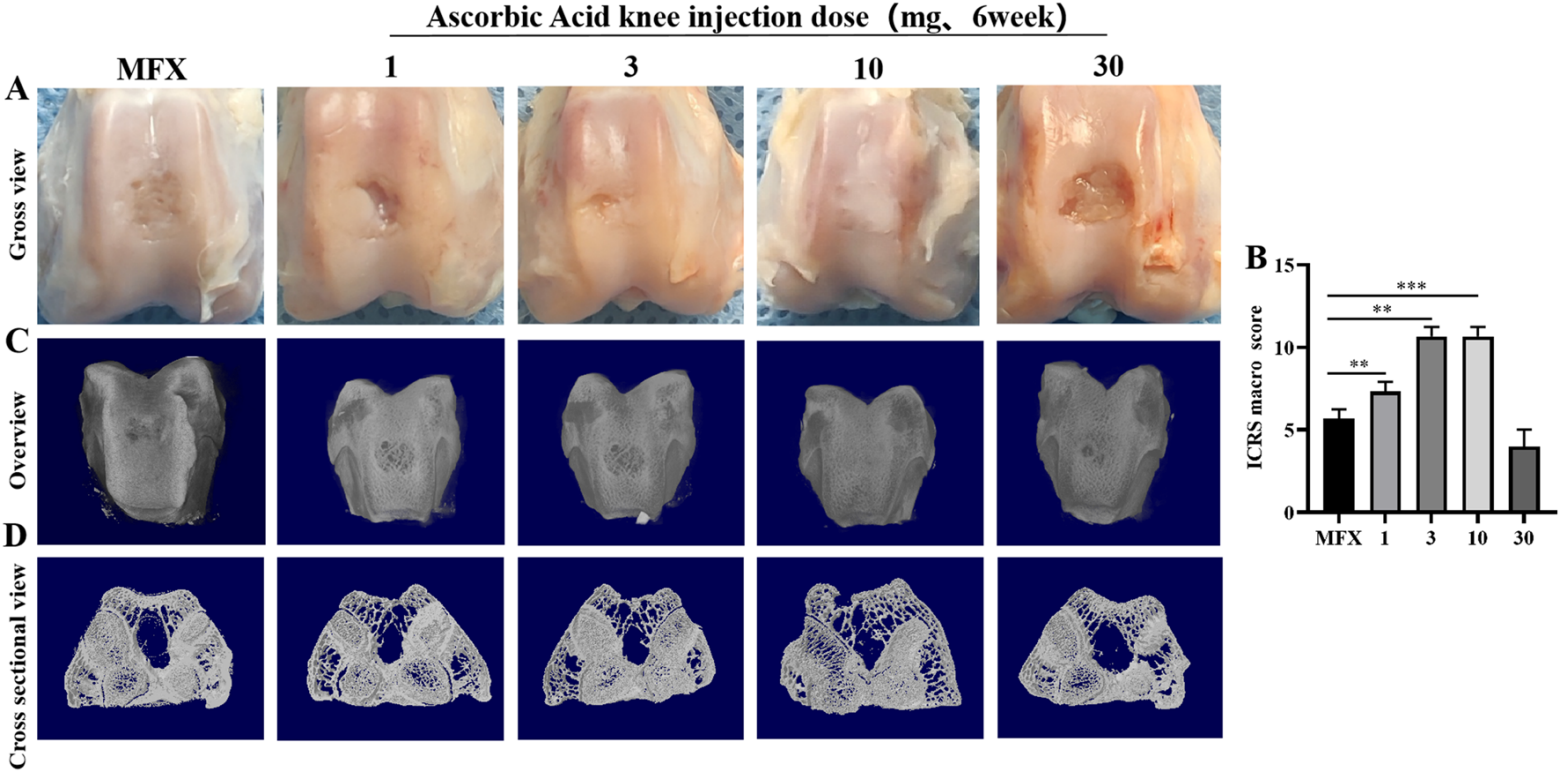
Micro-CT and gross images 6 weeks after surgery. (**A**, **B**) Gross image of osteochondral defect healing and the ICRS score. (**C**) Micro-CT 3D images. (**D**) Cross-sectional view of the centre of the osteochondral defect. Asterisks indicate statistical significance (*p < 0.05; **p < 0.01; ***p < 0.005).

Six weeks after surgery, gross observation revealed that the cartilage in the defect area in the 10 mg group had basically healed, and the colour was mostly the same as that of the surrounding host cartilage. The defect area was poorly healed in the MFX group and the 30 mg group, and a small amount of regenerated cartilage formed in the defect area. The 1 mg and 3 mg groups exhibited incomplete cartilage healing in the defect area, and the colour of the regenerated cartilage was basically the same as that of the surrounding cartilage (Fig. 1A). The 10 mg group had the highest ICRS score, as determined by macroscopic evaluation, and the score was significantly higher than that in the MFX group (Fig. 1B). Furthermore, we performed micro-CT, and the reconstructed images showed that the subchondral bone defect in the 10 mg group was basically healed. However, in the MFX group and the 1, 3, and 30 mg groups, the subchondral bone was not healed, and the healing of the microfracture hole was incomplete (Fig. 1C). A cross-sectional view of the middle area of the defect further showed that subchondral bone healing was the best in the 10 mg group. A small amount of bone trabeculae was observed in the subchondral bone in the 1 and 3 mg groups, and the MFX and 30 mg groups had more obvious subchondral bone defect areas and no significant bone trabecular regeneration (Fig. 1D).

### H&E staining and safranin O staining to evaluate cartilage repair 6 weeks after surgery

**Figure 2:**
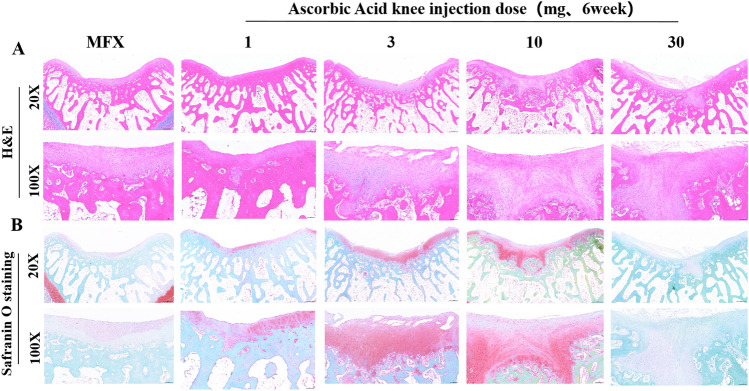
Haematoxylin and eosin (H&E) and Safranin O staining. (**A**) H&E Staining. (**B**) Safranin O staining of trochlear groove cartilage. Scale bar = 500 µm and 100 µm at 20× magnification and 100× magnification, respectively.

Six weeks after surgery, we performed H&E staining on the regenerated cartilage tissue. The nuclei were blue, and the cytoplasm was pink. The cartilage layers in groups were stained pink. At 20X magnification, the results showed that both the MFX group and the 1, 3, 10, and 30 mg groups had regenerated cartilage, and the subchondral bone did not fully heal. At 100× magnification, compared with that in the MFX group and the 1, 3, and 30 mg groups, the regenerated cartilage in the 10 mg group was the thickest, chondrocytes were evenly distributed in the cartilage layer, and the regenerated cartilage extended to the subchondral bone. A small amount of cartilage regeneration and fibrosis was observed on the surface of the cartilage in the MFX group and the 1, 3, and 30 mg groups, and the regenerated cartilage extended to subchondral bone in the 30 mg group (Fig. 2A).

We further stained glycosaminoglycans (GAGs) with safranin O. At 20× magnification, the regenerated cartilage in the MFX and 30 mg groups was mildly positive, while the cartilage surface in the MFX group was a light orange colour. In contrast, the regenerated cartilage of the 3 and 10 mg groups showed strong orange‒red staining, and the subchondral bone had not yet fully healed and was still orange‒red. At 100× magnification, the MFX group exhibited very weak orange‒red staining of the regenerated cartilage, and a small amount of fibrosis was observed on the surface of regenerated cartilage in the 1 mg and 3 mg groups. In the 10 mg group, the regenerated cartilage had a smooth and transparent structure on the surface, and in the 30 mg group, the regenerated cartilage extended to the subchondral bone, as shown by the very weak orange‒red staining (Fig. 2B).

### Immunohistochemical evaluation of the distribution of and changes in COL-I and COL-II 6 weeks after surgery

**Figure 3:**
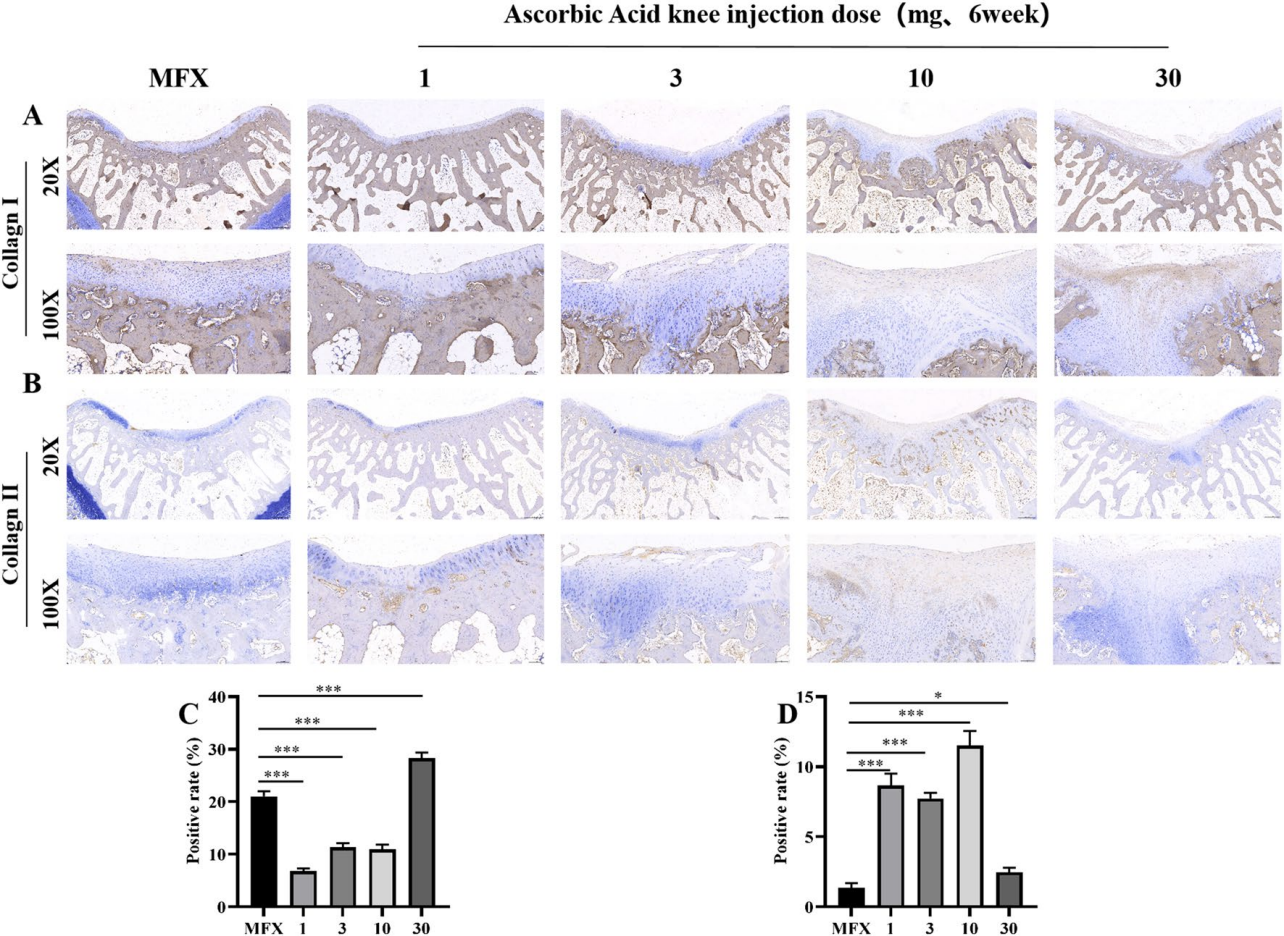
Immunohistochemical analysis of COL-I and COL-II. (**A**) Staining for type I collagen. (**B**) Staining for type II collagen. (**C**) Statistical chart showing the percentage of the type I collagen-positive area. (**D**) The chart shows the percentage of the type II collagen-positive area. Scale bars = 500 µm and 100 µm at 20× magnification and 100× magnification, respectively. Asterisks indicate statistical significance (*p < 0.05; **p < 0.01; ***p < 0.005).

Six weeks after surgery, we examined the expression level of COL-I, which is related to fibrocartilage. Brown indicates type I collagen, and at 20× magnification, only a small amount of COL-I was observed in the MFX group, while the level in the 30 mg group was relatively increased. Most COL-I was expressed in the subchondral bone matrix. The 1, 3, and 10 mg groups exhibited low or even no expression in regenerated cartilage. At 100× magnification, a large amount of type 1 collagen was observed on the surface and subchondral bone of the regenerated cartilage in the MFX and 30 mg groups, while the 1, 3, and 10 mg groups exhibited collagen in the subchondral bone area (Fig. 3A, C). We used immunohistochemistry (IHC) to examine type II collagen. Brown indicates COL-II, and at 20X magnification, the COL-II-positive areas in the 1, 3, and 10 mg groups were mostly located in the regenerated subchondral bone. The surface of the cartilage in the MFX group and 30 mg group was mostly negative. At 100× magnification, the surface of the cartilage in the MFX and 30 mg groups was mostly negative, and a small amount of COL-II was observed on the surface of the regenerated cartilage and subchondral bone in the 1 and 3 mg groups. Compared with the MFX group, the 10 mg group had higher levels of COL-II (Fig. 3B, D).

### Immunohistochemical evaluation of the distribution of and changes in COL-III 6 weeks after surgery

**Figure 4:**
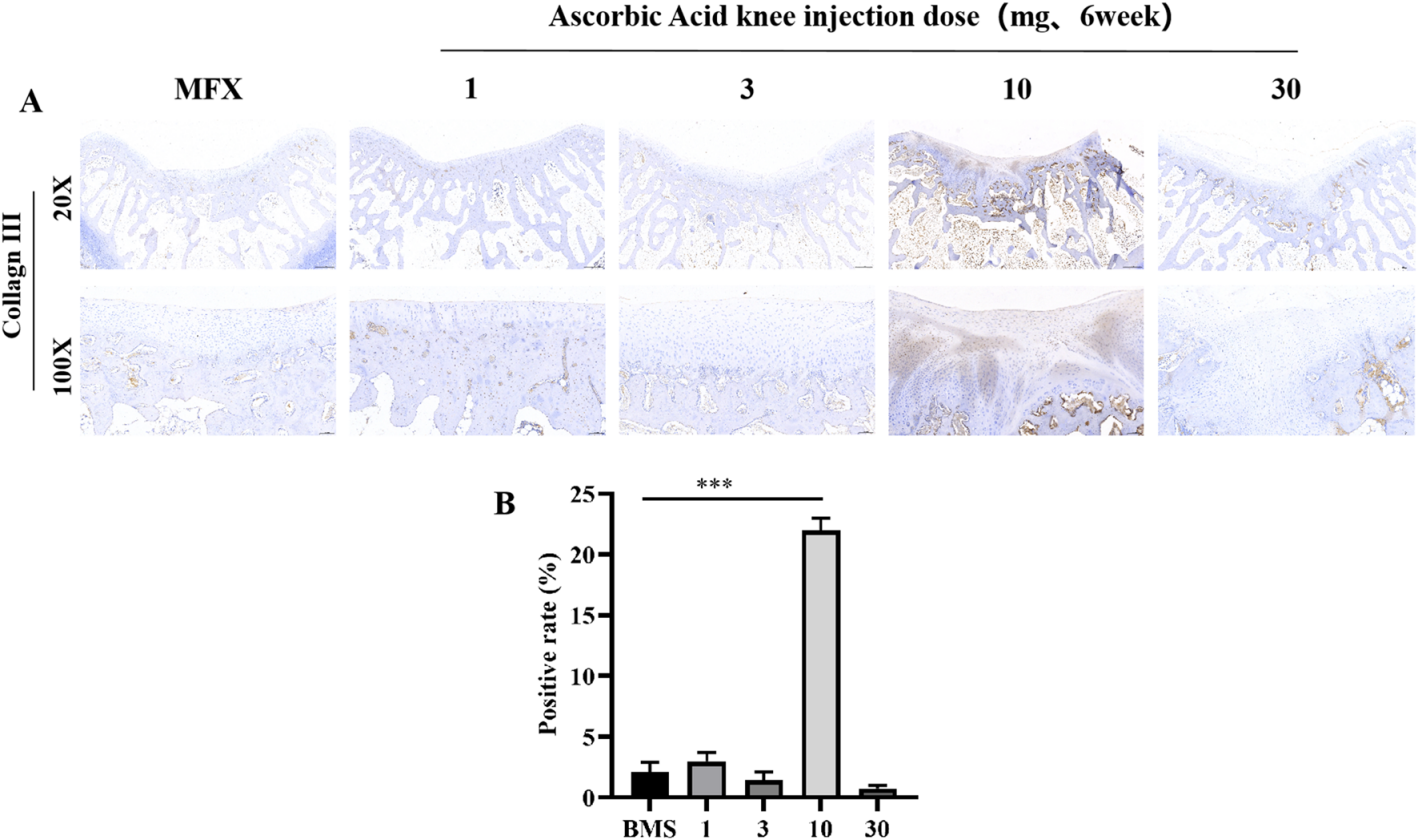
Immunohistochemical analysis of COL-III. (**A**) Staining for type III collagen. (**B**) Statistical chart showing the positive rate of type III collagen. Scale bars = 500 µm and 100 µm at 20× magnification and 100× magnification, respectively. Asterisks indicate statistical significance (*p < 0.05; **p < 0.01; ***p < 0.005).

At 20× magnification, large COL-III-positive areas were observed in the regenerated cartilage and subchondral bone in the 10 mg group, and a small amount of type III collagen was observed in the subchondral bone in the MFX group and the 1, 3, and 30 mg groups. At 100× magnification, type III collagen was expressed in the regenerated cartilage of the trochlear groove in the 10 mg group, while low or even no type III collagen was expressed in the regenerated cartilage in the MFX group and the 1, 3, and 30 mg groups (Fig. 4A, B).

### Gross observations and ICRS scores 12 weeks after surgery

**Figure 5:**
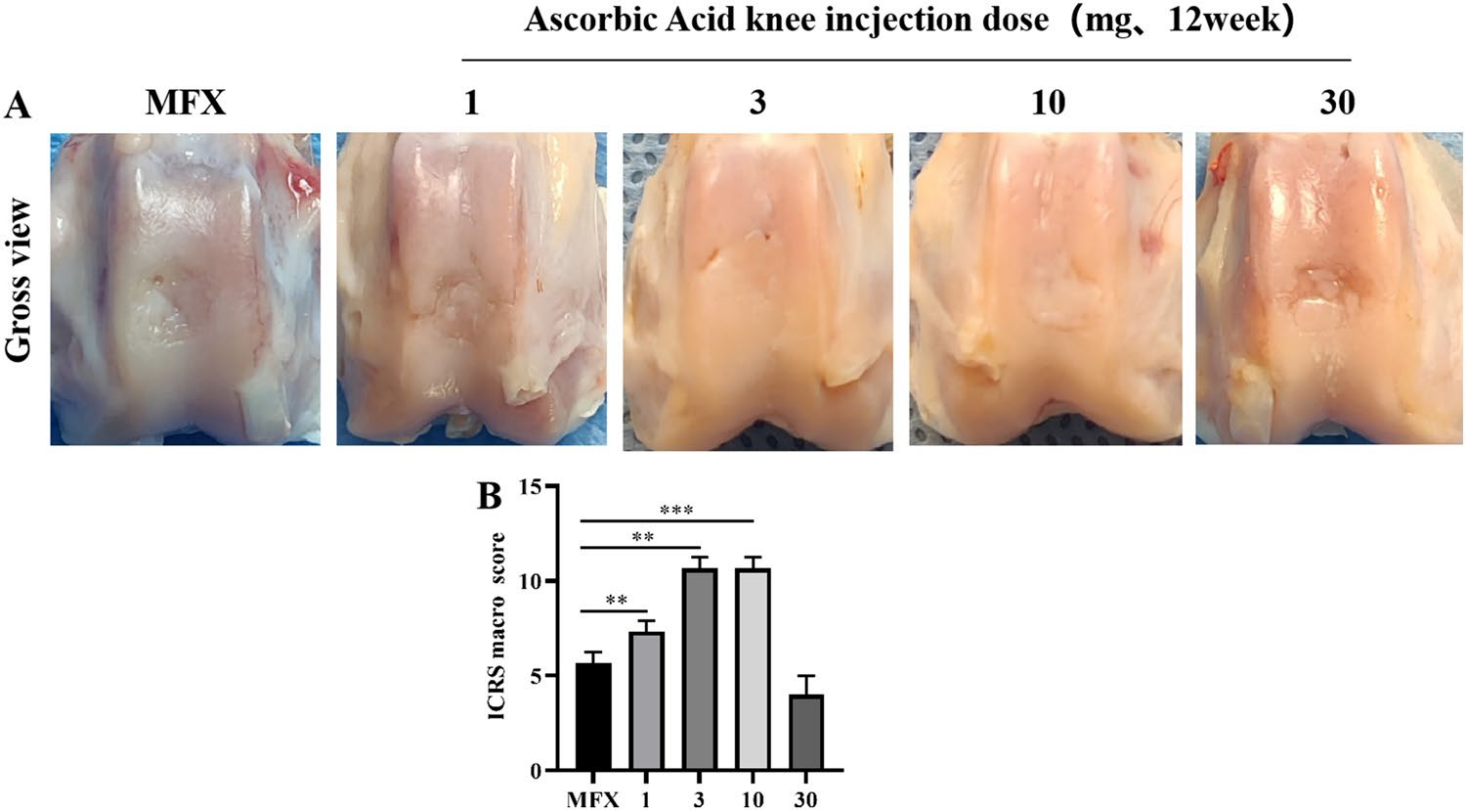
Gross images 6 weeks after surgery. (**A**) Gross image of osteochondral defect healing. (**B**) ICRS score. Asterisks indicate statistical significance (*p < 0.05; **p < 0.01; ***p < 0.005).

Twelve weeks after surgery, the cartilage in 10 mg group had basically healed, and the colour was mostly the same as that of the surrounding cartilage. There was a significant difference in colour between the regenerated cartilage in the defect area and the surrounding cartilage in the MFX group. The defect area was partially healed in the 1 mg, 3 mg, and 30 mg groups, and poor healing was observed in the 1 mg and 30 mg groups (Fig. 5A). Macroscopic evaluation showed that the 3 mg and 10 mg groups had significantly higher ICRS scores than the MFX group (Fig. 5B).

### H&E staining and safranin O staining to evaluate cartilage repair 12 weeks after surgery

Twelve weeks after surgery, H&E staining was performed at 20× magnification.

**Figure 6:**
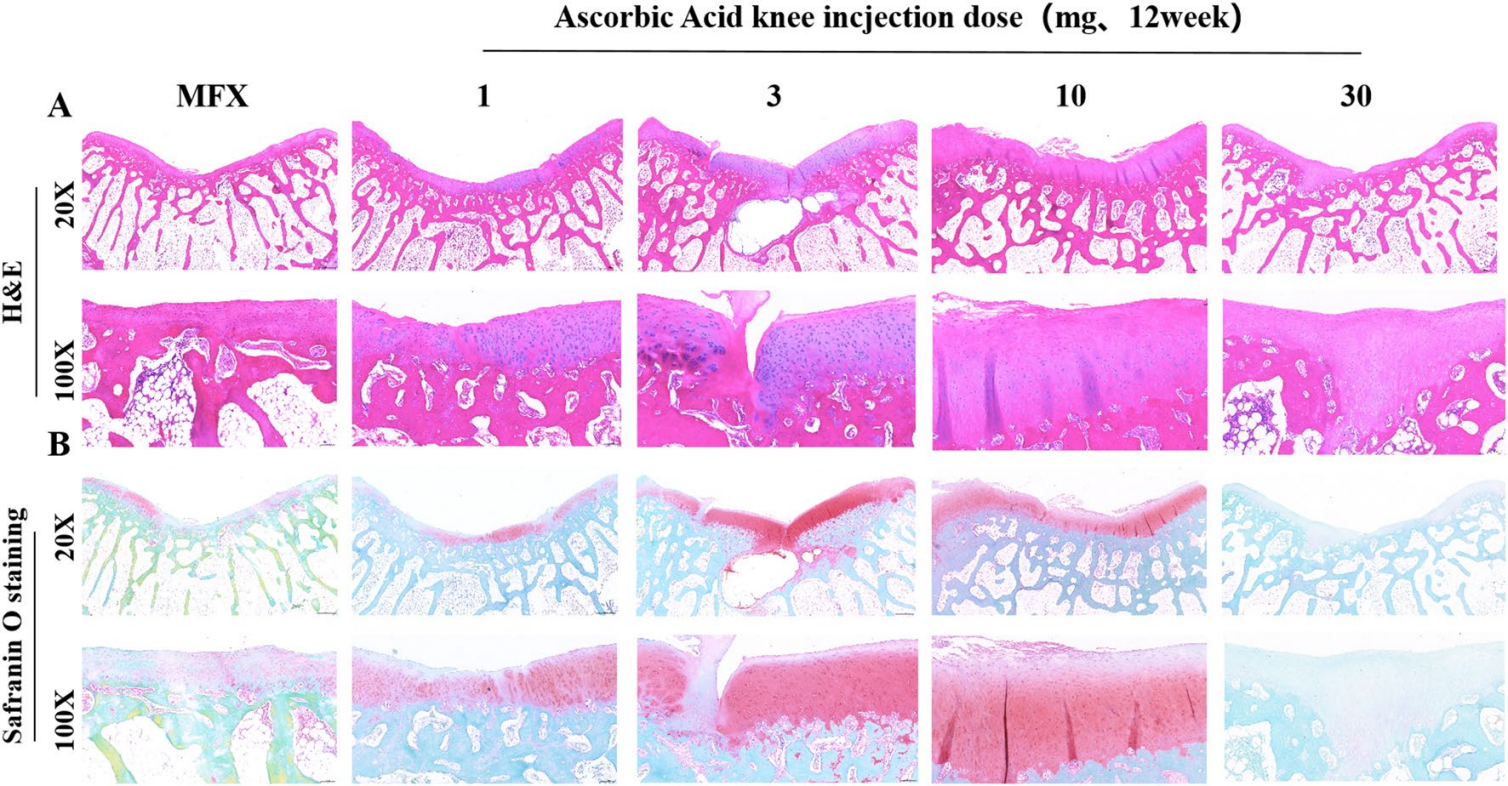
H&E and Safranin O staining (**A**) H&E staining. (**B**) Safranin O staining of the cochlear groove cartilage. Scale bars = 500 µm and 100 µm at 20× magnification and 100× magnification, respectively.

The subchondral bone was healed in all groups except for the 30 mg group, and a cavity formed by the subchondral bone was visible in the 3 mg group. At 100× magnification, the regenerated cartilage in the 10 mg group was the thickest and showed natural cartilage morphology. A small amount of cartilage regeneration was observed in the MFX group and 30 mg group, and a small amount of fibrosis was observed on the surface of the cartilage. The 1 mg and 3 mg groups exhibited extensive cartilage regeneration, a small amount of fibrosis on the surface of the cartilage, and an uneven distribution of chondrocytes (Fig. 6A). Safranin O staining revealed that at 20× magnification, the MFX group and the 1 mg and 30 mg groups showed mild Safranin O staining of regenerated cartilage. Compared to that in the MFX group, the regenerated cartilage in the 3 mg and 10 mg groups exhibited strong orange‒red staining. At 100× magnification, the MFX group exhibited very weak orange‒red staining of regenerated cartilage. The 1 mg group showed a small amount of fibrosis on the surface regenerated cartilage in the 3 mg and 10 mg groups was transparent, and regenerated cartilage showed no connection between regenerated cartilage and normal cartilage in the 3 mg group. Regenerated cartilage extended to the subchondral bone in the 30 mg group. The regenerated cartilage part showed very weak orange‒red staining (Fig. 6B).

### Immunohistochemical evaluation of the distribution of and changes in COL-I and COL-II 12 weeks after surgery

**Figure 7:**
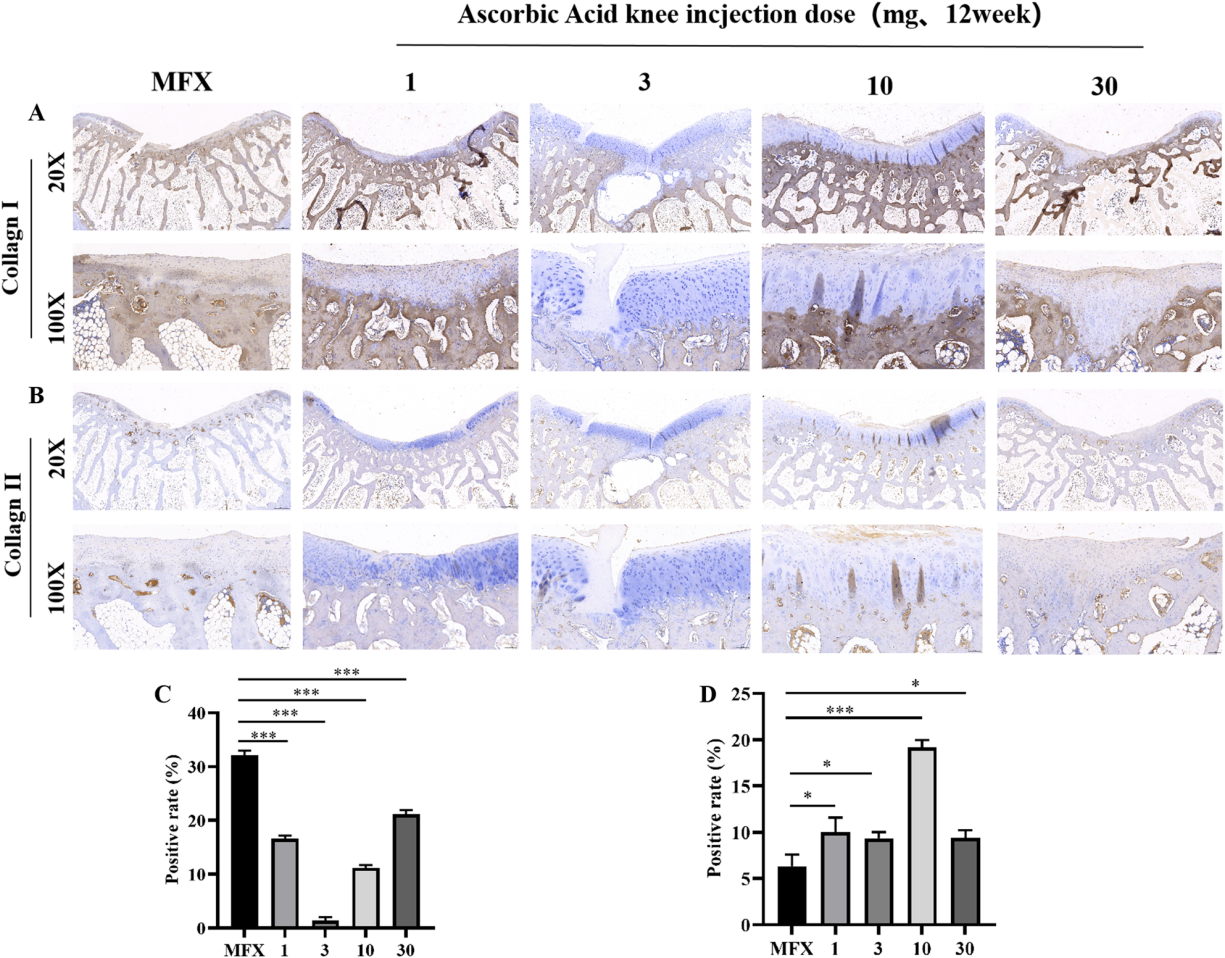
Immunohistochemical analysis of COL-I and COL-II. (**A**) Staining for type I collagen. (**B**) Staining for type II collagen. (**C**) Statistical chart showing the positive rate of type I collagen expression. (**D**) The chart shows the rate of type II collagen expression. Scale bars = 500 µm and 100 µm at 20× magnification and 100× magnification, respectively. Asterisks indicate statistical significance (*p < 0.05; **p < 0.01; ***p < 0.005).

Type 1 collagen staining revealed that at 20× magnification, a large amount of COL-I collagen was observed on the surface of regenerated cartilage in the MFX group and 30 mg group. The 1, 3, and 10 mg groups exhibited low expression in regenerated cartilage. At 100× magnification, a large amount of type 1 collagen was observed on the surface and subchondral bone of the regenerated cartilage in the MFX group and 30 mg group, and a small amount of type 1 collagen was expressed on the surface and subchondral bone of the regenerated cartilage in the 1 and 10 mg groups. Moreover, the 3 mg group showed no type 1 collagen on the surface of the regenerated cartilage or subchondral bone (Fig. 7A, C). Immunohistochemical analysis of type II collagen showed that at 20× magnification, large COL-II-positive areas were observed on the surface of the regenerated cartilage and subchondral bone in response to 10 mg. The COL-II-positive areas in the MFX, 1 mg, 3 mg, and 30 mg groups were mainly located in the subchondral bone. At 100× magnification, the surface of the cartilage in the MFX and 1 mg groups was mostly negative. A small amount of type II collagen was observed on the surface of the regenerated cartilage and subchondral bone in the 3 and 30 mg groups. Compared to that in the MFX group, a large amount of type II collagen was expressed in the 10 mg group (Fig. 7B, D).

### Immunohistochemical evaluation of the distribution of and changes in COL-III 12 weeks after surgery

**Figure 8:**
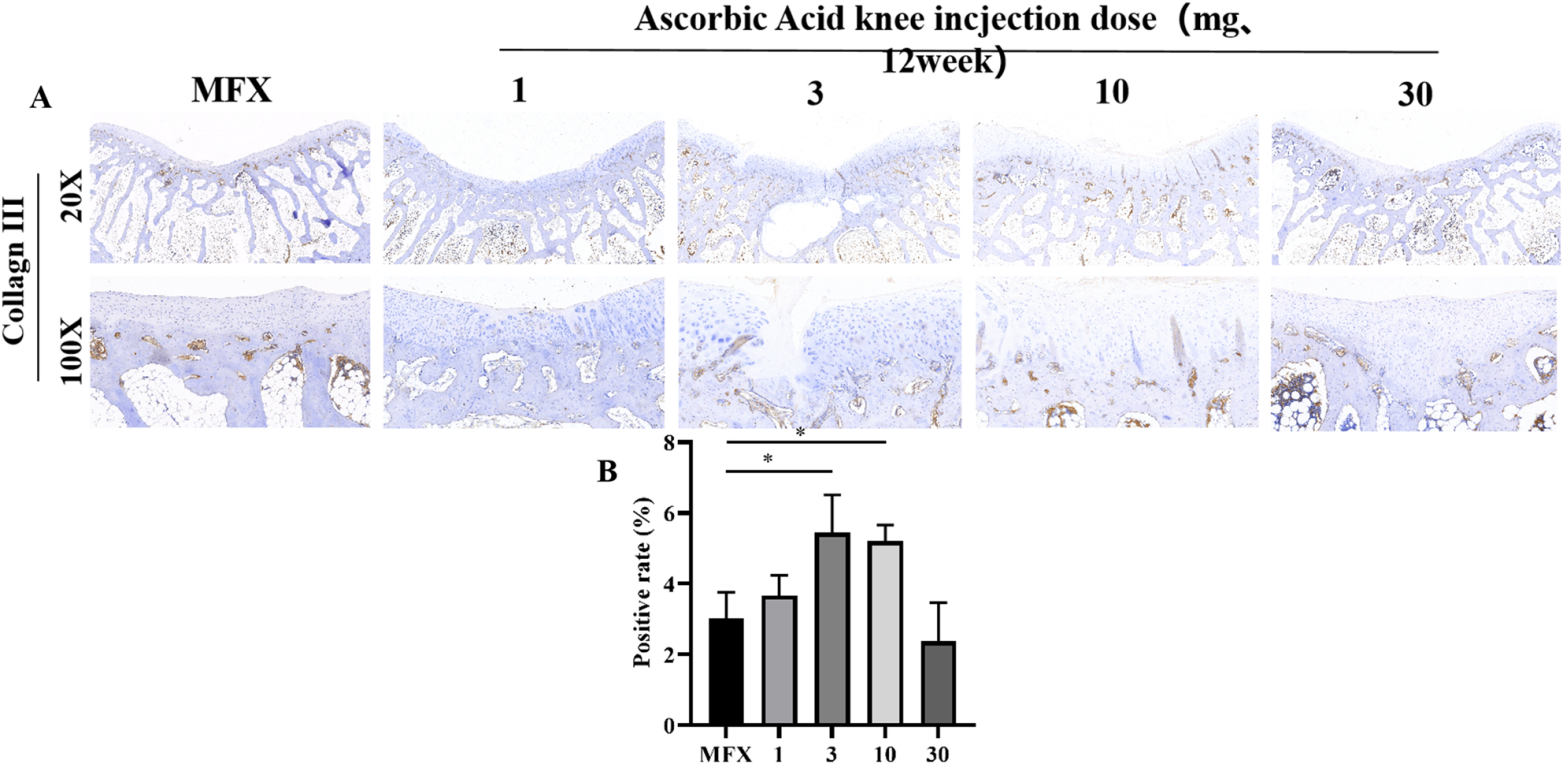
Immunohistochemical analysis of COL-III. (**A**) Staining of type III collagen. (**B**) Statistical chart showing the positive rate of type III collagen. Scale bars = 500 µm and 100 µm at 20× magnification and 100× magnification, respectively. Asterisks indicate statistical significance (*p < 0.05; **p < 0.01; ***p < 0.005).

At 20× magnification, low expression of COL-III was observed in the subchondral bone in the 3 and 10 mg groups, and low or no expression of COL-III was observed in the subchondral bone in the MFX group and the 1, 3, and 30 mg groups. At 100× magnification, the regenerated cartilage in the 3 and 10 mg groups expressed a small amount of COL-III. The MFX group and 1 and 30 mg groups showed low or even no expression in regenerated cartilage (Fig. 8A, B).

### Effect of AA injection on TGF-β1, AKT/Nrf2, and VEGF

**Figure 9:**
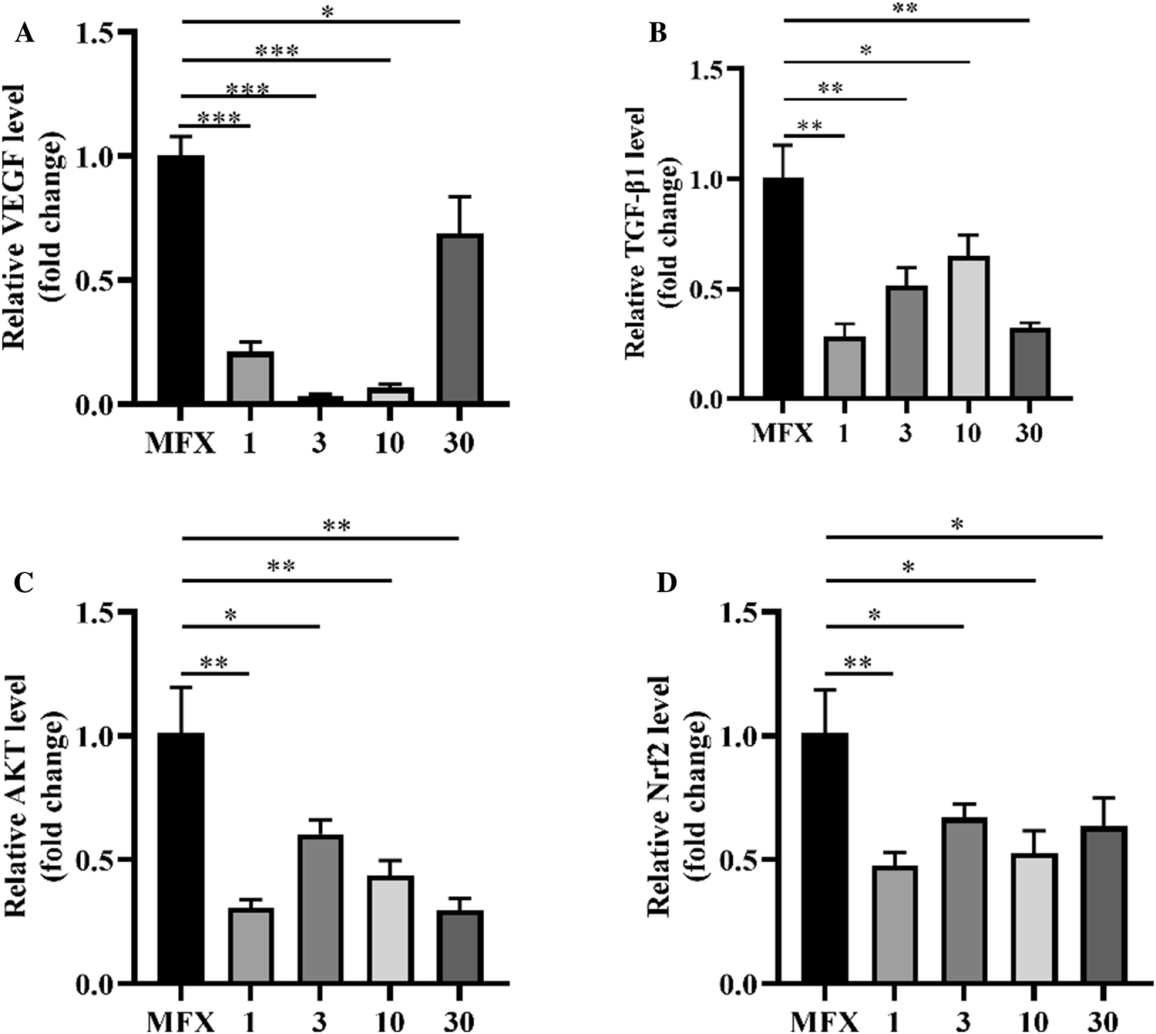
RT‒qPCR analysis of TGF-β1, AKT/Nrf2, and VEGF expression. (**A**) The expression of VEGF. (**B**) The expression of TGF-β1. (**C**) The expression of AKT. (**D**) The expression of Nrf2. Asterisks indicate statistical significance (*p < 0.05; **p < 0.01; ***p < 0.005).

We examined the expression of TGF-β1, AKT/Nrf2, and VEGF in the fat pad using RT‒qPCR. Compared with that in the MFX group, injection of AA significantly downregulated the mRNA expression of TGF-β1, AKT, Nrf2, and VEGF (Fig. 9A–D).

## Discussion

In this study, we showed that intra-articular injection of AA after MFX stimulation of bone marrow mesenchymal stem cells improved cartilage repair in a rabbit model, which was related to the promotion of hyaline cartilage formation by AA. In addition, after the injection of four concentrations of AA (1 mg/ml, 3 mg/ml, 10 mg/ml, and 30 mg/ml), 10 mg/ml AA significantly improved cartilage repair. Joint cavity injection of AA downregulated the expression of TGF-β1 and VEGF, thereby inhibiting the formation of fibrocartilage.

AA is an essential vitamin that participates in various biochemical reactions and plays a crucial role in maintaining growth^37^. AA as a drug approved by Food and Drug Administration, is currently widely used^38^. Moreover, AA can be replenished by the human body, mainly from green vegetables, citrus fruits, and potatoes. The recommended daily dose depends on factors such as age, sex, and smoking status^39^. AA deficiency is associated with a range of orthopaedic diseases, such as the development of degenerative intervertebral disc disease in elderly individuals^40^, skeletal dysplasia, osteoporosis^41^, and the development of osteoarthritis^42^. Research has shown that AA can enhance the intestinal absorption of iron, reducing iron (Fe^3+^) to a ferrous state (Fe^2+^), releasing Fe^2+^ from transporters to promote binding to ferrous ion receptors on MSCs, and iron is a known cofactor necessary for collagen synthesis, further contributing to maintaining musculoskeletal health^43^. AA functions as a key cofactor in various metabolic pathways, and cofactors are crucial for muscle and bone development and repair, as well as for collagen synthesis^43^. Moreover, vitamins play a crucial role in chondrogenic differentiation and extracellular matrix synthesis. In addition, AA promotes cell growth by increasing DNA synthesis and the differentiation of mesenchymal stem cells into various cell types, such as adipocytes, osteoblasts, myoblasts, and chondrocytes^44,45^. However, studies of AA combined with MFX have not yet been reported. Our study showed that the administration of 10 mg AA/knee could effectively promote the formation of type 2 collagen and promote cartilage repair. These findings may be beneficial for further clinical use of MFX combined with AA therapy.

After literature search showed that many drugs are currently available that can be combined with MFX technology. We found that at 6 weeks, 10 mg/ml AA was superior to bevacizumab according to gross observation, micro-CT, and histological staining^20^, and overall healing was similar to the effect of^18^ intra-articular injection of 1 mg and 10 mg losartan. At 12 weeks, macroscopic observation, ICRS scores, and histological staining showed that 10 mg/ml AA was superior to PRP^46^ and the small molecule compound bergenin^47^ for treating osteochondral defects. Six months after surgery, rhfGF-18 incompletely healed cartilage defects. Previous studies have reported that intra-articular injection of 3 mg/ml AA has the best therapeutic effect on osteoarthritis. However, in this study, gross observation, histological staining, and immunohistochemistry confirmed that 10 mg/ml was superior to 3 mg/ml at 6 and 12 weeks. This difference may be due to the release of BMSCs into the joint cavity after MFX surgery, which leads to a decrease in the AA concentration due to the release of blood cells. The co-factors in AA directly participate in the healing of surgical site wounds, resulting in decreased effects on BMSCs. AA enters the bone marrow cavity through microfracture holes, resulting in a shorter duration. Malicev et al.^48^ reported that AA concentrations higher than 1.136 mM could lead to human chondrocyte death. In our study, 30 mg/ml had a significant inhibitory effect on cartilage repair, which was further supported by the significant increase in histological staining and immunohistochemical expression of type 1 collagen.

AA can promote cartilage repair through various mechanisms. Previous studies have shown that AA can inhibit the expression of TGF^48^, inhibit the formation of fibrocartilage, and promote the regeneration of hyaline cartilage. AKT is an important cell survival signalling pathway and a key regulator of the Nrf-2 signalling pathway. AKT/Nrf-2 inhibits the production of ROS, indirectly promoting the synthesis of type II collagen and the formation of glycosaminoglycans^49,50^. VEGF has a damaging effect on cartilage, and inhibiting VEGF expression reduces angiogenesis and the progression of osteoarthritis and induces key factors in stem cell cartilage differentiationy^51^. Therefore, we examined the mRNA expression of TGF, AKT/Nrf2, and VEGF and found that AA injection significantly decreased the mRNA expression of TGF and VEGF. Overall, MFX combined with AA treatment significantly downregulated the expression of the chondrogenic genes TGF and VEGF, promoting the formation of hyaline chondrocytes.

The results of this study showed that three injections of 10 mg/ml AA after surgery significantly improved cartilage repair mediated by MFX technology by increasing the formation of hyaline proteins, such as cartilage. Translating these encouraging results into clinical applications requires injection during surgery and then two more injections after surgery. Previous studies have shown that AA promotes the formation of transparent cartilage through numerous mechanisms. If confirmed in large animal and human studies in the future, this AA injection method could provide a simple solution for MFX-mediated cartilage regeneration, further promoting the widespread application of enhanced microfracture treatment.

## Data Availability

All datasets presented in this study are included in the article.
